# 2-(1,2,3,4-Tetra­hydro-9*H*-carbazol-1-yl­idene)propane­dinitrile

**DOI:** 10.1107/S1600536810042649

**Published:** 2010-10-30

**Authors:** R. Velmurugan, M. Sekar, A. Chandramohan, P. Ramesh, M. N. Ponnuswamy

**Affiliations:** a Post Graduate and Research Department of Chemistry, Sri Ramakrishna Mission Vidyalaya College of Arts and Science, Coimbatore 641 020, India; b Centre of Advanced Study in Crystallography and Biophysics, University of Madras, Guindy Campus, Chennai 600 025, India

## Abstract

In the title compound, C_15_H_11_N_3_, the cyclo­hexene ring adopts a sofa conformation. An intra­molecular N—H⋯N hydrogen bond generates an *S*(7) ring motif. In the crystal, mol­ecules are linked by inter­molecular N—H⋯N, C—H⋯N and C—H⋯π inter­actions into a three-dimensional network.

## Related literature

For the biological activity of carbazole derivatives, see: Shufen *et al.* (1995 ▶); Magnus *et al.* (1992 ▶); Abraham (1975 ▶); Saxton (1983 ▶); Phillipson & Zenk (1980 ▶); Bergman & Pelcman (1990 ▶); Kirtikar & Basu (1933 ▶); Chakraborty *et al.* (1973 ▶). For puckering parameters, see: Cremer & Pople (1975 ▶). For asymmetry parameters, see: Nardelli (1983 ▶). For hydrogen-bond motifs, see: Bernstein *et al.* (1995 ▶).
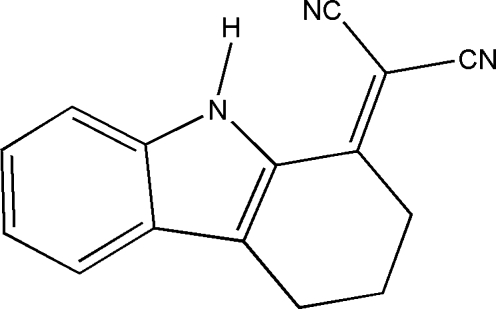


## Experimental

### 

#### Crystal data

C_15_H_11_N_3_
*M* *_r_* = 233.27
Triclinic, 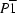

*a* = 7.7631 (10) Å
*b* = 8.0003 (10) Å
*c* = 9.8933 (13) Å
α = 87.461 (8)°
β = 82.392 (8)°
γ = 75.038 (7)°
*V* = 588.35 (13) Å^3^
*Z* = 2
Mo *K*α radiation
μ = 0.08 mm^−1^
*T* = 293 K
0.20 × 0.18 × 0.17 mm

#### Data collection

Bruker SMART APEXII area-detector diffractometer
Absorption correction: multi-scan (*SADABS*; Bruker, 2008 ▶) *T*_min_ = 0.984, *T*_max_ = 0.986
10589 measured reflections
2924 independent reflections
2339 reflections with *I* > 2σ(*I*)
*R*_int_ = 0.039

#### Refinement

*R*[*F* ^2^ > 2σ(*F* ^2^)] = 0.041
*wR*(*F* ^2^) = 0.130
*S* = 1.06
2924 reflections
168 parameters
2 restraints
H atoms treated by a mixture of independent and constrained refinement
Δρ_max_ = 0.23 e Å^−3^
Δρ_min_ = −0.15 e Å^−3^

### 

Data collection: *APEX2* (Bruker, 2008 ▶); cell refinement: *SAINT* (Bruker, 2008 ▶); data reduction: *SAINT*; program(s) used to solve structure: *SHELXS97* (Sheldrick, 2008 ▶); program(s) used to refine structure: *SHELXL97* (Sheldrick, 2008 ▶); molecular graphics: *ORTEP-3* (Farrugia, 1997 ▶); software used to prepare material for publication: *SHELXL97* and *PLATON* (Spek, 2009 ▶).

## Supplementary Material

Crystal structure: contains datablocks global, I. DOI: 10.1107/S1600536810042649/bt5359sup1.cif

Structure factors: contains datablocks I. DOI: 10.1107/S1600536810042649/bt5359Isup2.hkl

Additional supplementary materials:  crystallographic information; 3D view; checkCIF report

## Figures and Tables

**Table 1:** Hydrogen-bond geometry (Å, °) *Cg*3 is the centroid of the C8–C13 ring.

*D*—H⋯*A*	*D*—H	H⋯*A*	*D*⋯*A*	*D*—H⋯*A*
N1—H1⋯N16	0.885 (17)	2.623 (17)	3.3314 (16)	137.8 (13)
N1—H1⋯N16^i^	0.885 (17)	2.279 (17)	3.0656 (17)	148.1 (14)
C12—H12⋯N16^i^	0.93	2.62	3.3254 (19)	133
C4—H4*B*⋯*Cg*3^ii^	0.97	2.86	3.6950 (15)	145

Symmetry codes: (i) 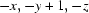; (ii) 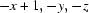.

**Fig. 1.**
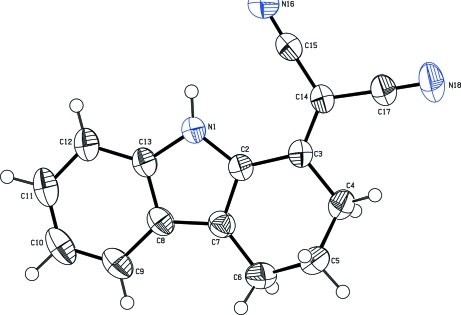
The molecular structure of the title compound, showing the atomic numbering and displacement ellipsoids drawn at the 50% probability level.

**Fig. 2.**
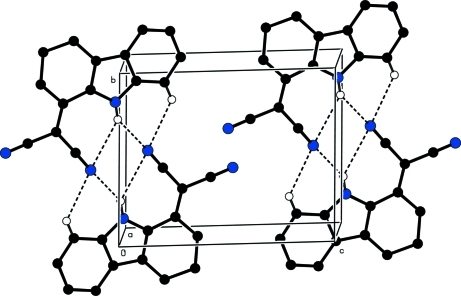
The crystal packing of the title compound. H atoms not involved in hydrogen bonding (dashed lines) have been omitted for clarity.

## supplementary crystallographic information

### Comment

Carbazole alkaloids obtained from naturally occurring sources have been the subject of extensive research, mainly because of their widespread application in traditional medicine (Bergman & Pelcman, 1990; Kirtikar & Basu, 1933). Aminocarbazoles are widely used as intermediates for the preparation of carbazole-based synthetic dyes, agrochemicals, pharmaceuticals, light-sensitive materials (Shufen *et al.*, 1995). Tetrahydrocarbazole systems are present in the framework of a number of indole-type alkaloids of biological interest (Magnus *et al.*, 1992; Abraham, 1975; Saxton, 1983; Phillipson *et al.*, 1980). These types of compounds possess significant antibiotic, anti-carcinogenic, antiviral and anti-inflammatory properties (Chakraborty *et al.*, 1973). Against this background and to ascertain the molecular structure and conformation, the X-ray structure determination of the title compound has been carried out.

The *ORTEP* plot of the molecule is shown in Fig. 1. The cyclohexane ring in the carbazole ring system adopts sofa conformation with the puckering parameters (Cremer & Pople, 1975) and the asymmetry parameters (Nardelli, 1983) are: q_2_ =0.378 (1) Å, q_3_ = -0.274 (1) Å, φ_2_ = 353.6 (2)° and Δ_s_(C2 & C5)= 6.11 (13)°. The sum of the bond angles around N1 [359.6°] is in accordance with *sp^2^* hybridization. The bond angles of (C14—C15—N16) 178.3 (1)° and (C14—C17—N18) 178.9 (2)° show linear character of the cyano group, a feature observed in carbonitrile compounds.

The crystal packing reveals that symmetry-related molecules are linked through a network by C—H···N, N—H···N, C—H···π and π···π types of intra and intermolecular interactions. The intramolecular N1—H1···N16 hydrogen bond generates a S(7) ring motif. The molecules at (*x*, *y*, *z*) and (-*x*, -*y* + 1, -*z*) are linked by C12—H12···N16 hydrogen bonds into cyclic centrosymmetric *R*_2_^2^(18) dimer. The dimers are cross-linked *via* C—H···π intermolecular interactions.

### Experimental

A mixture of 1-oxo-1,2,3,4-tetrahydrocarbazole (7.5 mmol), and melanonitrile (7.5 mmol), ammonium acetate (0.57 g, 8.125 mmol) and acetic acid (1.5 ml, 24.75 mmol) in 12.5 ml of toluene was stirred at 105°C for five 5 h. On cooling the precipitate that formed was filtered off, washed with hexane (20 ml) and dried at 100°C to give a crude product of 1-(dicyanomethylene) -2,3,4-tetrahydrocarbazole. The crystals of the title compound suitable for single XRD analysis were obtained by the slow evaporation method by using dichloroethane as solvent at room temperature.

### Refinement

N-bound H atom was located in a difference map and refined isotropically. C-bound H atoms were positioned geometrically (C–H = 0.93–0.97 Å) and allowed to ride on their parent atoms, with *U*_iso_(H) = 1.2*U*_eq_(C) for all H atoms. The components of the anisotropic displacement parameters of (C14-C15) and (C14-C17) in the direction of the bond between them were restrained to be equal within an effective standard deviation of 0.001.

### Crystal data

**Table:**

C_15_H_11_N_3_	*Z* = 2
*M**_r_* = 233.27	*F*(000) = 244
Triclinic, *P*1	*D*_x_ = 1.317 Mg m^−^^3^
Hall symbol: -P 1	Mo *K*α radiation, λ = 0.71073 Å
*a* = 7.7631 (10) Å	Cell parameters from 1654 reflections
*b* = 8.0003 (10) Å	θ = 2.1–28.4°
*c* = 9.8933 (13) Å	µ = 0.08 mm^−^^1^
α = 87.461 (8)°	*T* = 293 K
β = 82.392 (8)°	Block, colorless
γ = 75.038 (7)°	0.20 × 0.18 × 0.17 mm
*V* = 588.35 (13) Å^3^	

### Data collection

**Table:**

Bruker SMART APEXII area-detector diffractometer	2924 independent reflections
Radiation source: fine-focus sealed tube	2339 reflections with *I* > 2σ(*I*)
graphite	*R*_int_ = 0.039
ω and φ scans	θ_max_ = 28.4°, θ_min_ = 2.1°
Absorption correction: multi-scan (*SADABS*; Bruker, 2008)	*h* = −10→10
*T*_min_ = 0.984, *T*_max_ = 0.986	*k* = −10→10
10589 measured reflections	*l* = −12→13

### Refinement

**Table:**

Refinement on *F*^2^	Secondary atom site location: difference Fourier map
Least-squares matrix: full	Hydrogen site location: inferred from neighbouring sites
*R*[*F*^2^ > 2σ(*F*^2^)] = 0.041	H atoms treated by a mixture of independent and constrained refinement
*wR*(*F*^2^) = 0.130	*w* = 1/[σ^2^(*F*_o_^2^) + (0.0748*P*)^2^ + 0.0374*P*] where *P* = (*F*_o_^2^ + 2*F*_c_^2^)/3
*S* = 1.06	(Δ/σ)_max_ = 0.003
2924 reflections	Δρ_max_ = 0.23 e Å^−^^3^
168 parameters	Δρ_min_ = −0.15 e Å^−^^3^
2 restraints	Extinction correction: *SHELXL97* (Sheldrick, 2008), Fc^*^=kFc[1+0.001xFc^2^λ^3^/sin(2θ)]^-1/4^
Primary atom site location: structure-invariant direct methods	Extinction coefficient: 0.038 (8)

### Special details

**Table:**

Geometry. All e.s.d.'s (except the e.s.d. in the dihedral angle between two l.s. planes) are estimated using the full covariance matrix. The cell e.s.d.'s are taken into account individually in the estimation of e.s.d.'s in distances, angles and torsion angles; correlations between e.s.d.'s in cell parameters are only used when they are defined by crystal symmetry. An approximate (isotropic) treatment of cell e.s.d.'s is used for estimating e.s.d.'s involving l.s. planes.
Refinement. Refinement of *F*^2^ against ALL reflections. The weighted *R*-factor *wR* and goodness of fit *S* are based on *F*^2^, conventional *R*-factors *R* are based on *F*, with *F* set to zero for negative *F*^2^. The threshold expression of *F*^2^ > σ(*F*^2^) is used only for calculating *R*-factors(gt) *etc*. and is not relevant to the choice of reflections for refinement. *R*-factors based on *F*^2^ are statistically about twice as large as those based on *F*, and *R*- factors based on ALL data will be even larger.

### Fractional atomic coordinates and isotropic or equivalent isotropic displacement parameters (Å^2^)

**Table:**

	*x*	*y*	*z*	*U*_iso_*/*U*_eq_	
N1	0.18203 (13)	0.14402 (13)	0.01388 (9)	0.0404 (2)	
H1	0.118 (2)	0.252 (2)	0.0038 (17)	0.068 (4)*	
C2	0.25866 (13)	0.06198 (14)	0.12645 (10)	0.0375 (2)	
C3	0.26818 (13)	0.13570 (14)	0.25321 (10)	0.0380 (3)	
C4	0.36565 (17)	0.01146 (17)	0.35406 (13)	0.0491 (3)	
H4A	0.3179	0.0527	0.4456	0.059*	
H4B	0.4918	0.0112	0.3398	0.059*	
C5	0.35020 (18)	−0.17242 (16)	0.34360 (13)	0.0532 (3)	
H5A	0.2257	−0.1756	0.3670	0.064*	
H5B	0.4204	−0.2461	0.4078	0.064*	
C6	0.41700 (17)	−0.23982 (16)	0.20031 (13)	0.0518 (3)	
H6A	0.5464	−0.2582	0.1830	0.062*	
H6B	0.3889	−0.3498	0.1910	0.062*	
C7	0.32980 (14)	−0.11254 (14)	0.09931 (11)	0.0412 (3)	
C8	0.29475 (14)	−0.13856 (15)	−0.03433 (12)	0.0430 (3)	
C9	0.32862 (18)	−0.28496 (18)	−0.11570 (14)	0.0559 (3)	
H9	0.3900	−0.3926	−0.0849	0.067*	
C10	0.26989 (19)	−0.2670 (2)	−0.24143 (15)	0.0621 (4)	
H10	0.2916	−0.3636	−0.2963	0.075*	
C11	0.17753 (18)	−0.1052 (2)	−0.28854 (13)	0.0561 (3)	
H11	0.1390	−0.0972	−0.3743	0.067*	
C12	0.14204 (16)	0.04168 (17)	−0.21264 (11)	0.0486 (3)	
H12	0.0815	0.1487	−0.2451	0.058*	
C13	0.20122 (14)	0.02303 (15)	−0.08386 (11)	0.0407 (3)	
C14	0.20193 (15)	0.30577 (15)	0.28668 (11)	0.0436 (3)	
C15	0.11508 (19)	0.43809 (15)	0.19901 (13)	0.0519 (3)	
N16	0.0464 (2)	0.54705 (16)	0.13082 (13)	0.0753 (4)	
C17	0.22133 (19)	0.36577 (18)	0.41741 (14)	0.0575 (3)	
N18	0.2367 (2)	0.4156 (2)	0.51944 (15)	0.0918 (5)	

### Atomic displacement parameters (Å^2^)

**Table:**

	*U*^11^	*U*^22^	*U*^33^	*U*^12^	*U*^13^	*U*^23^
N1	0.0490 (5)	0.0377 (5)	0.0343 (5)	−0.0091 (4)	−0.0075 (4)	−0.0041 (4)
C2	0.0391 (5)	0.0380 (5)	0.0360 (5)	−0.0103 (4)	−0.0051 (4)	−0.0024 (4)
C3	0.0375 (5)	0.0424 (6)	0.0356 (5)	−0.0120 (4)	−0.0056 (4)	−0.0026 (4)
C4	0.0505 (6)	0.0526 (7)	0.0441 (6)	−0.0081 (5)	−0.0158 (5)	0.0000 (5)
C5	0.0597 (7)	0.0476 (7)	0.0496 (7)	−0.0054 (5)	−0.0160 (5)	0.0062 (5)
C6	0.0517 (6)	0.0408 (6)	0.0591 (7)	−0.0023 (5)	−0.0114 (5)	−0.0020 (5)
C7	0.0392 (5)	0.0395 (6)	0.0435 (6)	−0.0073 (4)	−0.0035 (4)	−0.0055 (4)
C8	0.0418 (5)	0.0427 (6)	0.0433 (6)	−0.0102 (4)	0.0009 (4)	−0.0103 (5)
C9	0.0591 (7)	0.0477 (7)	0.0585 (8)	−0.0100 (5)	0.0009 (6)	−0.0191 (6)
C10	0.0647 (8)	0.0658 (9)	0.0577 (8)	−0.0225 (7)	0.0074 (6)	−0.0300 (7)
C11	0.0576 (7)	0.0769 (9)	0.0397 (6)	−0.0278 (7)	0.0000 (5)	−0.0173 (6)
C12	0.0521 (6)	0.0592 (7)	0.0371 (6)	−0.0183 (5)	−0.0043 (5)	−0.0075 (5)
C13	0.0418 (5)	0.0458 (6)	0.0357 (5)	−0.0140 (4)	−0.0010 (4)	−0.0075 (4)
C14	0.0511 (6)	0.0433 (6)	0.0391 (5)	−0.0133 (5)	−0.0107 (4)	−0.0061 (4)
C15	0.0725 (8)	0.0386 (6)	0.0466 (6)	−0.0132 (5)	−0.0150 (5)	−0.0068 (5)
N16	0.1197 (11)	0.0434 (6)	0.0624 (8)	−0.0100 (7)	−0.0318 (7)	0.0008 (6)
C17	0.0693 (8)	0.0528 (7)	0.0502 (7)	−0.0071 (6)	−0.0194 (6)	−0.0137 (6)
N18	0.1192 (12)	0.0874 (10)	0.0674 (9)	−0.0048 (9)	−0.0392 (8)	−0.0328 (8)

### Geometric parameters (Å, °)

**Table:**

N1—C13	1.3655 (14)	C7—C8	1.4193 (16)
N1—C2	1.3906 (14)	C8—C9	1.3991 (16)
N1—H1	0.885 (17)	C8—C13	1.4114 (17)
C2—C7	1.3865 (15)	C9—C10	1.370 (2)
C2—C3	1.4278 (15)	C9—H9	0.9300
C3—C14	1.3634 (16)	C10—C11	1.401 (2)
C3—C4	1.5098 (16)	C10—H10	0.9300
C4—C5	1.5156 (19)	C11—C12	1.3698 (18)
C4—H4A	0.9700	C11—H11	0.9300
C4—H4B	0.9700	C12—C13	1.3997 (16)
C5—C6	1.5159 (18)	C12—H12	0.9300
C5—H5A	0.9700	C14—C15	1.4257 (17)
C5—H5B	0.9700	C14—C17	1.4386 (16)
C6—C7	1.4921 (16)	C15—N16	1.1413 (17)
C6—H6A	0.9700	C17—N18	1.1334 (17)
C6—H6B	0.9700		
			
C13—N1—C2	108.44 (9)	C2—C7—C8	106.56 (10)
C13—N1—H1	121.1 (11)	C2—C7—C6	123.35 (10)
C2—N1—H1	130.1 (11)	C8—C7—C6	130.04 (10)
C7—C2—N1	109.30 (9)	C9—C8—C13	119.21 (12)
C7—C2—C3	122.29 (10)	C9—C8—C7	133.37 (12)
N1—C2—C3	128.42 (10)	C13—C8—C7	107.37 (10)
C14—C3—C2	125.47 (10)	C10—C9—C8	118.88 (13)
C14—C3—C4	119.15 (10)	C10—C9—H9	120.6
C2—C3—C4	115.36 (10)	C8—C9—H9	120.6
C3—C4—C5	113.84 (10)	C9—C10—C11	120.89 (12)
C3—C4—H4A	108.8	C9—C10—H10	119.6
C5—C4—H4A	108.8	C11—C10—H10	119.6
C3—C4—H4B	108.8	C12—C11—C10	122.23 (12)
C5—C4—H4B	108.8	C12—C11—H11	118.9
H4A—C4—H4B	107.7	C10—C11—H11	118.9
C4—C5—C6	110.58 (11)	C11—C12—C13	116.83 (13)
C4—C5—H5A	109.5	C11—C12—H12	121.6
C6—C5—H5A	109.5	C13—C12—H12	121.6
C4—C5—H5B	109.5	N1—C13—C12	129.73 (11)
C6—C5—H5B	109.5	N1—C13—C8	108.31 (10)
H5A—C5—H5B	108.1	C12—C13—C8	121.95 (11)
C7—C6—C5	109.78 (10)	C3—C14—C15	125.01 (10)
C7—C6—H6A	109.7	C3—C14—C17	120.54 (11)
C5—C6—H6A	109.7	C15—C14—C17	114.43 (11)
C7—C6—H6B	109.7	N16—C15—C14	178.28 (13)
C5—C6—H6B	109.7	N18—C17—C14	178.87 (17)
H6A—C6—H6B	108.2		
			
C13—N1—C2—C7	0.92 (12)	C7—C8—C9—C10	177.34 (12)
C13—N1—C2—C3	−179.03 (10)	C8—C9—C10—C11	0.1 (2)
C7—C2—C3—C14	179.75 (10)	C9—C10—C11—C12	0.2 (2)
N1—C2—C3—C14	−0.31 (19)	C10—C11—C12—C13	−0.53 (18)
C7—C2—C3—C4	1.66 (15)	C2—N1—C13—C12	178.09 (11)
N1—C2—C3—C4	−178.40 (10)	C2—N1—C13—C8	−1.16 (12)
C14—C3—C4—C5	150.75 (11)	C11—C12—C13—N1	−178.53 (11)
C2—C3—C4—C5	−31.03 (14)	C11—C12—C13—C8	0.64 (17)
C3—C4—C5—C6	56.40 (14)	C9—C8—C13—N1	178.93 (10)
C4—C5—C6—C7	−50.35 (13)	C7—C8—C13—N1	0.97 (12)
N1—C2—C7—C8	−0.30 (12)	C9—C8—C13—C12	−0.40 (17)
C3—C2—C7—C8	179.65 (9)	C7—C8—C13—C12	−178.35 (10)
N1—C2—C7—C6	−177.95 (10)	C2—C3—C14—C15	−1.19 (19)
C3—C2—C7—C6	2.00 (17)	C4—C3—C14—C15	176.84 (11)
C5—C6—C7—C2	23.15 (15)	C2—C3—C14—C17	−179.26 (11)
C5—C6—C7—C8	−153.90 (12)	C4—C3—C14—C17	−1.23 (17)
C2—C7—C8—C9	−177.95 (12)	C3—C14—C15—N16	−161 (5)
C6—C7—C8—C9	−0.5 (2)	C17—C14—C15—N16	17 (5)
C2—C7—C8—C13	−0.40 (12)	C3—C14—C17—N18	149 (9)
C6—C7—C8—C13	177.03 (11)	C15—C14—C17—N18	−30 (9)
C13—C8—C9—C10	0.02 (18)		

### Hydrogen-bond geometry (Å, °)

**Table:**

Cg3 is the centroid of the C8–C13 ring.

**Table:**

*D*—H···*A*	*D*—H	H···*A*	*D*···*A*	*D*—H···*A*
N1—H1···N16	0.885 (17)	2.623 (17)	3.3314 (16)	137.8 (13)
N1—H1···N16^i^	0.885 (17)	2.279 (17)	3.0656 (17)	148.1 (14)
C12—H12···N16^i^	0.93	2.62	3.3254 (19)	133
C4—H4B···Cg3^ii^	0.97	2.86	3.6950 (15)	145

Symmetry codes:  (i) −*x*, −*y*+1, −*z*; (ii) −*x*+1, −*y*, −*z*.
